# Prevention, Risk Exposure, and Knowledge of Monkeypox in Occupational Settings: A Scoping Review

**DOI:** 10.3390/tropicalmed7100276

**Published:** 2022-09-29

**Authors:** Lucrezia Ginevra Lulli, Antonio Baldassarre, Nicola Mucci, Giulio Arcangeli

**Affiliations:** Department of Experimental and Clinical Medicine, University of Florence, 50134 Florence, Italy

**Keywords:** monkeypox, global health, zoonosis, outbreak, occupational health, healthcare worker, prevention, vaccination, occupational exposure, education

## Abstract

With ongoing climate change, which alters the conditions for pathogens and vectors, zoonotic diseases such as monkeypox virus will become a challenge and a great threat impacting global health in future decades. A current outbreak of monkeypox is occurring in over 125 countries, with a report of thousands of cases in countries where this virus has never appeared. Occupational exposure to the monkeypox virus has recently been identified as an issue of major concern for occupational health, especially in healthcare settings. A scoping review following the PRISMA guidelines was performed, aiming to analyze the effects that the current monkeypox outbreak has in workplaces, given the potential exposure of healthcare workers to the virus, the possible spread of the virus in occupational settings, and the preventive measures that are necessary to implement. At the end of the selection process, 21 studies were included in the review. Healthcare workers are considered at a high risk, and similar preventive measures to those adopted during the SARS-CoV-2 pandemic must be implemented in all healthcare settings. The main recommendations for preventing and managing monkeypox in occupational settings are the vaccination of exposed workers, the prompt identification and isolation of infected individuals, and good hygiene practices. Education and specific training are necessary in non-endemic countries to make healthcare workers able to recognize the disease and prevent further contagions. Although monkeypox seems unlikely to reach the pandemic spread of COVID-19, an approach to global health even to avoid future zoonotic epidemics is required by all stakeholders.

## 1. Introduction

The COVID-19 pandemic is not over yet, and monkeypox (MPX), a new—or better, an emerging—virus, is again threatening public health in several countries around the world. Monkeypox, as with smallpox, is a member of the Orthopoxvirus group (family Poxviridae, subfamily Chordopoxvirinae). There are two distinct clades (groups of similar organisms descended from a common ancestor) of monkeypox: the West African clade and the Congo Basin clade. Presumably occurring in sub-Saharan Africa for thousands of years, it was first isolated in a monkey colony in Copenhagen in 1958, hence the name, albeit nonhuman primates are not monkeypox virus reservoirs. Although the reservoir is unknown, the leading candidates are small rodents, such as squirrels, in the rain forests, mostly in western and central Africa. The first zoonotic transmission and human case was reported in a 9-month-old child from Zaire in 1970. A dramatic increase in the incidence in Africa since 2000 is thought to be due to the cessation of smallpox vaccination in 1980, which provided protection after 20 or more years [1,2,3,4]. Starting from May 2022, thousands of cases of monkeypox have been reported in countries where this virus has never appeared or has appeared sporadically for imported cases and a limited chain of transmission [5,6]. On the contrary, the current outbreak has involved 125 countries, of which 96 have not historically reported monkeypox [7]. The World Health Organization (WHO) has declared the current monkeypox outbreak a high priority and a global health emergency in July 2022. As of September 2022, over 57.000 cases have been confirmed worldwide since January 2022, with 18 deaths, while the European region is considered by WHO to be at a high risk [8]. Monkeypox is transmitted to humans through close contact with an infected animal or person through lesions, body fluids, respiratory droplets, or with material contaminated with the virus [9]. In the current monkeypox outbreak, transmission through skin and mucosal contact during sexual activities was the most commonly reported, and based on the European Centre for Disease Prevention and Control (ECDC)’s epidemiological assessment, the likelihood of MPX spreading in persons having multiple sexual partners in the European Union (EU) is considered high [10]. Possible high-risk groups may be very young children, pregnant women, and elderly or immunocompromised individuals among close contacts of MPX cases. The infection rate among household contacts (unvaccinated) has been estimated at around 10% [11]. Other populations that may be at a higher risk of contracting monkeypox are specific categories of workers, in particular those working in healthcare settings. In fact, during this outbreak, healthcare workers (HCWs) are often likely to be exposed to monkeypox patients. In addition, the misrecognition of the lesions of monkeypox—which can manifest in a non-typical way—increase the risk that occupational contacts can occur without the proper personal protective equipment (PPE). According to recent guidelines, the PPE requested are a disposable gown; disposable gloves; disposable shoes or boots covers; a Filtering Face Piece Class 2 respirator (FFP2), in compliance with the EU standard EN 149 or equivalent; and eye splash protection, in compliance with the EU standard EN 166 or equivalent [10]. In addition, workers in contact with animals that can host the monkeypox virus may be at risk for occupational exposure. As we learned from the COVID-19 pandemic, risk awareness, its assessment, and the implementation of preventive measures in living and working environments are crucial to protecting public health. In addition, the attitudes of workers toward the risk play a critical role for the implementation of preventive measures in the workplace. The monkeypox virus is a novel pathogen in most of the countries where it is currently spreading, and gathering knowledge of its possible transmission in workplaces is fundamental at this very moment of the outbreak. This review thus aims at analyzing the effects that the current monkeypox outbreak has in workplaces, given the potential exposure of healthcare workers to the virus, the possible spread of the virus in other occupational settings such as veterinarian activities, and the preventive measures that are necessary to implement. As a scoping review, the objective of this research is also to define the nature and extent of the research evidence regarding the monkeypox virus in occupational settings, including the relevant topics studied and the research gaps.

## 2. Materials and Methods

### 2.1. Objectives of the Review

At this very moment, guidelines have been provided by the public health authorities all over the world regarding the necessary measures to address the current outbreak, including several recommendations for healthcare workers. However, a specific focus on the monkeypox risk in occupational settings is missing. Therefore, this review aims to collect evidence from the literature and previous monkeypox outbreaks regarding three main questions:

- What is the occupational risk of contracting monkeypox, mainly in healthcare settings, but possibly also in other working sectors such as veterinarian environments?
- What are the preventive measures that should be implemented and were applied in previous monkeypox outbreaks to prevent contagion in working settings?
- Since the healthcare setting is the main high-risk working environment, what is the level of knowledge and attitude of healthcare workers toward the monkeypox virus?

### 2.2. Methods 

This review follows the PRISMA extension for scoping reviews (PRISMA-ScR) [12] and the PRISMA-S protocol [13]. The online search was initially performed on 11 August 2022, and was updated on 8 September 2022. The online databases searched included Pubmed, Scopus, Embase, and Web of Science. A manual search for the references of the most relevant papers was also performed. The search was conducted using the following PICO strategy:

- Population: workers
- Intervention: spread of monkeypox virus
- Comparison: not applicable
- Outcome: risk of exposure/preventive measures/knowledge and attitude

The ad hoc research strings are reported in Table 1.

No limits on the time of publication, language, or type of paper were set; non-peer-reviewed articles were excluded. International guidelines such as the WHO, CDC, and ECDC recommendations were excluded from the selection of papers, but are considered in the Discussion. Inclusion and exclusion criteria referred to the PICO strategy, including the type of population, disease, and outcomes. The results were screened through titles and abstracts and the final selection was made through the reading of full texts. Data were collected in a table, including reference, country, year of publication, the main outcome addressed, and a brief summary. Then, a synthetic and narrative summary of the papers selected was carried out.

## 3. Results

**Figure 1:**
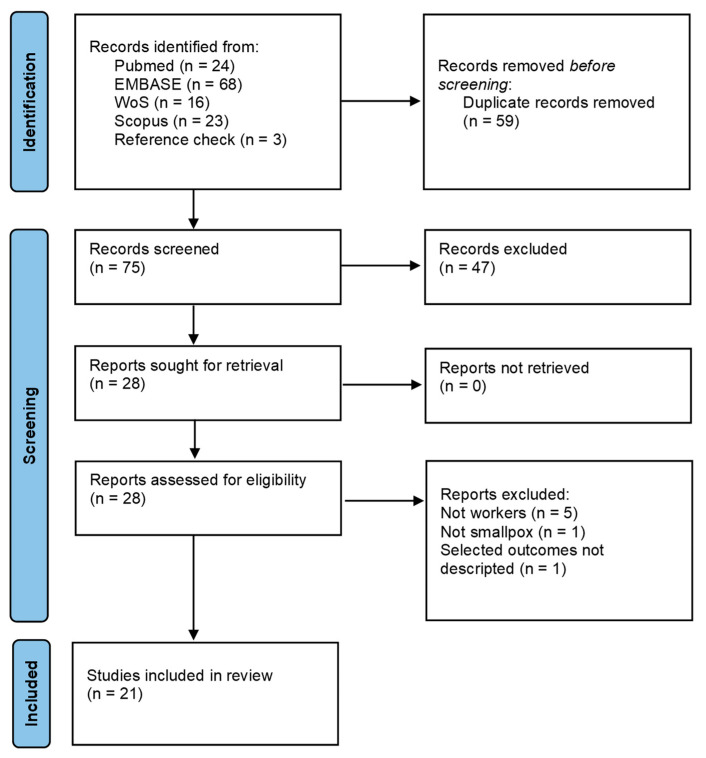
Flow chart of the selection process [14].

The research retrieved a total of 134 papers. At the end of the selection process, 21 studies were included in the review. The selection process is described in Figure 1.

Six studies were conducted in Europe (28%), five in the USA (24%), five in Africa (24%), and five (24%) in Asia. Fourteen studies (67%) were published before the current monkeypox outbreak. Table A1 in the Appendix A includes the list of selected papers and Table A2 provides the main preventive monkeypox guidelines in the four regions where the selected studies were conducted.

Four main topics were identified:

1. Risk of contagion in healthcare settings;
2. Preventive measures for contagion prevention in healthcare settings, including training and risk assessment;
3. Knowledge and attitudes of HCWs toward monkeypox;
4. Evidence for the prevention and knowledge of monkeypox in other occupational settings.

### 3.1. Risk of Contagion in Healthcare Settings

The evidence of occupational infections and a quantification of the risk of contagion in healthcare workers are mostly unknown, since the studies collecting evidence on these topics are anecdotal. Documented contagion in healthcare settings has been reported by Vaughan [15], before the current monkeypox outbreak. The infection occurred in a healthcare assistant wearing disposable aprons and gloves, almost two weeks after contact with an infected patient and nevertheless having received a post-exposure vaccination (>4 days after the contact). The only exposure risk identified was the changing of potentially contaminated bedding. Four other subjects of 134 identified among the healthcare assistant’s contacts became infected with monkeypox. A study dating back to 2005 [16] reported the surveillance data of 81 healthcare workers exposed to three monkeypox patients, with one of them requiring critical pediatric care. HCW exposure was defined as entrance into the immediate care area (2 m radius surrounding the patient) and protected exposure was defined as exposure with the use of personal protective equipment recommended for droplet precautions (gloves, gown, and a surgical or N95 mask). A total of 70% of HCWs had at least one unprotected exposure, but none reported symptoms consistent with the monkeypox illness. Another occupational transmission of monkeypox was described in the Central African Republic in 2016 [17], following a pediatric case. In fact, it was reported that five individuals outside the family of the child were infected with developed monkeypox: one hospital nurse, one doctor, one nurse who accompanied the patients during the transfer to the hospital, and two individuals who transported the patients to the hospital. Security measures were reinforced only after the first nurse became ill. In an effort to quantify the phenomenon of occupational infection, between 2010 and 2014, 1266 suspected monkeypox cases were investigated in the Tshuapa region in the Democratic Republic of Congo [18], of whom eleven worked as healthcare workers. Among the 699 confirmed cases, six confirmed cases of monkeypox among HCWs represented a proportion of 0.9% (range of 0.3–3.1% by year), yielding an estimated annual HCW incidence rate of 17.4/10,000, which is much more elevated than that of the general population. In more recent studies conducted in high-income countries [19,20,21,22], several HCWs were identified as contacts, but most were classified as low risk. Notably, three studies reported monkeypox cases during the COVID-19 pandemic and no secondary occupational infection was reported following the active surveillance. A recent study [23] assessed the viral load on surfaces in healthcare facilities where two patients affected by monkeypox were hospitalized: all surfaces directly touched by the patients’ hands showed viral contamination, with the highest loads detected in bathrooms. Monkeypox virus DNA was also found on the patient’s room surfaces, on fabrics used by patients, and also in the anterooms, even if with a lower viral load.

#### Risk Assessment of Monkeypox Exposure

Exposed workers should be evaluated regarding their risk and receive counselling on self-monitoring, isolation, and the prompt reporting of symptoms; aerosolizing procedures, such as shaking bed sheets, should be considered high risk. Exposed healthcare workers should undergo a 21-day active surveillance to cover the incubation period. Outpatient settings should also be provided, with adequate PPE given the current manifestations of the disease. Post-exposure vaccinations can be given with the available variola vaccines (VARV), ideally within four days of the exposure and up to fourteen days past exposure [19,20,24].

### 3.2. Preventive Measures in Healthcare Settings

#### 3.2.1. General Hygiene Measures

Following the recent outbreak, the centers of disease control, both American and European, as well as national Ministries of Health have provided guidelines on the protective measures to be followed in healthcare settings to reduce the risk of contagion and the spread of the virus. There is a large consensus that appropriate airborne-precautionary PPE is needed, including a fit-tested filtering facepiece respirator (for example, an N95 respirator) or a powered air-purifying respirator, gloves, and gowns [24,25]. A patient’s skin lesions should be covered with a bandage or gauze. Since poxviruses are very stable and may remain contagious in the environment, disinfectants should be used for the cleaning and disinfection of high-touch surfaces, and procedures (such as sweeping, dry dusting, or shaking bed linens) that may aerosolize virus particles should be avoided [26]. Linens should be removed carefully and washed at high heat or discarded. In a previous monkeypox outbreak in Nigeria, several preventive measures took place, including reserving an entire ward for the treatment of infected patients [27]. Generally, it is recommended to place the patients in dedicated rooms, if available with negative pressure [25,26]. In surgical settings, preventive measures such as those adopted for COVID-19 should be implemented. Aerosol-generating procedures and several surgical procedures (e.g., laparoscopic surgery, intubation) can generate aerosols infected with the virus. Therefore, there is a need to apply procedures to reduce the aerosol production during surgeries, such as rapid sequence intubation, limiting the use of equipment such as harmonic scalpels, utilizing smoke evacuation systems, and others [26].

#### 3.2.2. Training and Education

Education and training on recognizing the disease is crucial [24]. Recently, Koenig et al. [28] presented a framework tool to be used by emergency department policymakers, educators, and clinicians on the frontline. It is based on the “identify–isolate–inform” procedure and should especially help prehospital workers: patients are identified as potentially exposed or infected after an initial assessment of risk factors with associated signs and symptoms. Prehospital workers must immediately don personal protective equipment (PPE) and isolate infectious patients; the exchange of information with agency infection control offices must also take place as quickly as possible. In previous African monkeypox outbreaks [27,29], the implemented measures included workshops to inform and educate healthcare staff about monkeypox; clinicians were trained on the various aspects of standard precautions of infection control, the use of personal protective equipment, and healthcare waste management.

This section is divided by subheadings. It should provide a concise and precise description of the experimental results and their interpretation, as well as the experimental conclusions that can be drawn.

### 3.3. Knowledge and Attitudes of HCWs toward Monkeypox and Vaccination

One of the challenges in preventing monkeypox is the lack of knowledge about the disease among healthcare workers. In regions such as Europe or Southeast Asia, where cases of monkeypox never occurred until a few months ago, physicians may encounter some struggle in recognizing and treating monkeypox, which is a fundamental part of the prevention strategy. In a study conducted in Indonesia in 2020 among general practitioners [30], 10.0% and 36.5% of them had a good knowledge using an 80% and 70% cutoff point for the knowledge domain, respectively. Younger doctors had better knowledge, but the overall knowledge of monkeypox was low in all groups. A more recent study conducted among 163 general practitioners, public health doctors, and occupational physicians in Italy in May 2022 [31] demonstrated that the knowledge status for monkeypox infections was quite unsatisfying, with substantial knowledge gaps in all aspects of the disease and with a substantial overlooking of monkeypox as a pathogen, particularly when compared to SARS-CoV-2, TB, HIV, and HBV. However, the attitude toward vaccination was positive, despite concerns expressed by the WHO in 2019 about vaccine hesitation, which was ranked among the 10 threats to global health. In Indonesia, even before the COVID-19 pandemic, over 90% of doctors participating in a cross-sectional study about the attitude toward monkeypox vaccination were willing to be vaccinated [32].

### 3.4. Other Occupational Settings

In the literature research, the biological risk linked to the monkeypox virus was also identified in occupational settings dealing with pets and wildlife animals. Since monkeypox has a zoonotic transmission, veterinarians, pet staff, and other categories of workers with close contact with animals can be considered at high risk. In 2003, an outbreak of monkeypox virus infections occurred in the US, and this was the first outbreak recognized outside Africa. African rodents, which were identified as the primary carriers, had been housed with prairie dogs, which were subsequently distributed as household pets. Occupationally, transmitted infections occurred in twelve veterinary staff, two pet store employees, and two animal distributors. Working directly with animal care, being involved in prairie dog examination, caring for an animal within 6 feet of an ill prairie dog, and feeding an ill prairie dog were all activities associated with a higher risk of contracting the disease [33]. Human contact with wildlife is a major pathway for emerging and endemic infectious diseases, with 62% of all newly emerging infectious diseases being zoonotic, and monkeypox is one of them. In working sectors dealing with wildlife, such as the bushmeat trade, which is often illegal but very common in several African countries, knowledge regarding zoonosis and safety practices are fundamental to reduce the risk of exposure. In a study conducted regarding attitudes, practices, and zoonoses awareness of hunters and meat cookers in Uganda in 2020, only 62% of those interviewed acknowledged monkeypox for its zoonotic potential [34]. With a rise in the number of monkeypox cases, infected workers could contaminate the workplace, not only in healthcare settings, but also in offices. A revelation of viral load was made in an office where a patient worked during the prodromal phase of the illness. Low, but still present, traces of the virus were detected on a few superficies (3/34), demonstrating that contamination, although very limited, can occur in workplace environments, and accordingly, appropriate cleaning and decontamination measures should be considered in such situations [35].

## 4. Discussion

With ongoing climate change, which alters the conditions for pathogens and vectors, zoonotic diseases such as the monkeypox virus will become a challenge and a great threat impacting global health in future decades [36]. In addition, COVID-19 was originally born as a zoonotic infection, transmitted to humans working in the Wuhan market [37,38]. Hemorrhagic fever viruses, hantaviruses, arenaviruses, arboviruses, and zoonotic influenza viruses have the potential to cause severe epidemics [39]. As with monkeypox, zoonotic diseases are common in Africa. The paucity of information, lack of knowledge, limited resources, and cultural traditions make the African continent a hotspot for vector-borne and zoonotic viral diseases, which may spread globally [40]. This is what happened with monkeypox virus, which was probably exported from Nigeria around 2018–2019 [41] and subsequently spread to over 100 countries. The diffusion of monkeypox in several countries outside Africa is a major public health concern. Although it is quite unlikely that this outbreak will reach the proportion of a pandemic [42], several regions such as Central and Western Europe have been declared at a high risk and monkeypox has also become a public health concern in high-income countries [8]. Therefore, concerns are raising about the safety of workers that may be occupationally exposed to the virus, especially in light of the lessons learned from COVID-19 [43]. The findings of this scoping review show that occupational transmission is possible and has occurred several times in healthcare and veterinarian settings during past outbreaks in Africa and in the US. However, the papers describing such chains of contagion are limited. Monkeypox has been endemic for decades in some regions of central Africa, but has been neglected by high-income countries and by the health community in general [44]. In remote areas where monkeypox is endemic, the surveillance system of contagious diseases is lacking or struggles with a poor health system and a reporting system that is even worse. Therefore, it can be argued that there may have been an underestimate of occupational contagions. In addition, given the zoonotic transmission pathway, which before this outbreak was the prevalent one [45], an underestimated diffusion between other professionals can be hypothesized, including all the workers who engage in close contact with animals. In fact, while the animal reservoir is unknown, small mammals (e.g., rope and sun squirrels, giant-pouched rats, and African dormice) are thought to maintain the virus in the environments of West and Central Africa [7]. To what extent monkeypox can spread to domestic and farm animals is still unknown, but concerns have been raised [46], and in the future, workers in contact with specific species of animals may also be at risk. However, at this moment, according to our findings, the work category which is most exposed to occupational monkeypox risk is healthcare workers. The European Centre for Disease Prevention and Control (ECDC) has stated that healthcare workers in close contact with monkeypox patients without adequate PPE are at a high risk, and this is confirmed by the findings in the literature, where several infections have occurred before the recognition of the disease and the wearing of proper equipment. The findings of this review also show that the monkeypox virus can remain on surfaces for a long time, although its potential for infection is unknown, as reported for SARS-CoV-2 [47]. In order to prevent occupational contagion, it is mandatory to provide PPE to healthcare facilities, to educate operators on recognizing the lesions typical of the disease, and to continue to pay the necessary attention to hand and working environment hygiene [48,49]. These are the pillars of the preventive measures to be implemented in workplaces such as healthcare settings. Since the disease forms of this outbreak are mild [50], these interventions should take place not only in hospitals, but also in outpatient clinics, such as general practitioner clinics, with a widespread diffusion in the territory. On one hand, over two and a half years of a COVID-19 pandemic has brought mental and physical exhaustion to healthcare workers dealing with incessant “waves” of COVID-19, but on the other, there are some advantages to having experienced such a long struggle with the Sars-Cov-2 virus. In fact, healthcare workers are now confident in wearing the PPE requested for monkeypox protection, which is similar to that used for COVID-19 protection, and with all the other preventive measures enhanced during the pandemic. Moreover, contact tracing, active surveillance, searching for cases, and risk assessment have been practiced for a long time and have proven to be effective at reducing the spread of the virus [51,52]. In this regard, hospital and clinic management as well as occupational physicians are meant to play a fundamental role, as with that assumed during the COVID-19 pandemic [53]. A precise risk assessment process is indeed able to stop the chain of contagion in workplaces and outside, and to protect the workers’ health through secondary prevention measures. According to WHO [54], healthcare workers who have occupational exposures to patients with monkeypox or possibly contaminated materials do not need to be excluded from work duty if asymptomatic, but should actively monitor for symptoms for 21 days following the exposure. In addition, they should be instructed not to work with vulnerable patients during this period. Another important measure in this regard is vaccination. Available vaccines, based on a live, attenuated vaccinia virus (e.g., JYNNEOS and IMVANEX), have shown substantial effectiveness against MPX that has been estimated at around 85% [55,56]. Where vaccines are available, post-exposure vaccination (ideally within four days of exposure, and up to 14 days in the absence of symptoms) is recommended for healthcare workers to prevent the onset of disease or mitigate disease severity. A primary vaccination can be recommended for healthcare workers at a high risk of exposure, laboratory personnel working with Orthopoxviruses, clinical laboratory personnel performing diagnostic testing, and outbreak response team members [57]. Programs of sensibilization towards the vaccination of healthcare workers, as previously implemented with COVID, are therefore useful, given the fact that healthcare workers may also show vaccine hesitancy [58,59]. Implementing all these measures keeps the risk of occupational transmission low, at least in countries where monkeypox is not currently endemic [60]. Moreover, the findings of this review underline that the lack of knowledge of the disease, as well as its underestimated risk in healthcare workers, can be a concerning issue in the public and for occupational prevention strategies. If the disease is not promptly recognized, the chances of it spreading dramatically increase. Training and education are therefore fundamental preventive measures, not only to protect workers and public health, but also to reduce the mental and stress burden that an infectious outbreak is known to have on workers [61,62,63]. In this regard, the main national and international stakeholders, such as medical associations, public health authorities, and governments, are in charge of providing a specific formation to healthcare workers, not only to treat patients, but also to protect their own safety and health. Finally, this research has also identified some major gaps in the literature. The reports of occupational exposure and infections are limited and mainly anecdotal, and information about other working sectors besides healthcare is almost missing. Studies on the knowledge and attitudes of workers towards monkeypox are restricted to national (or even regional) dimensions and are not able to depict a clear picture of the ongoing situation. More research is needed in order to have a clearer framework of the occupational risk due to monkeypox and the effectiveness of prevention measures. Table 2 summarizes the key findings of the review along with their practical implications.

This review, as with all studies, presents with several limitations and points of strength. Regarding the former, it is a scoping review; thus, it does not have the rigor of a systematic review. This review, however, tries to comply with the highest standards of reporting evidence, following the PRISMA protocol. Although the research was the most accurate possible, some relevant papers may have been excluded not on purpose. Finally, the evidence retrieved is limited, and above all, the papers selected often had anecdotal or opinion forms (case report, case series, or editorial) that lowered the level of evidence in this review. This makes it unfeasible, in our opinion, to perform a systematic review at this moment. Regarding the latter, to our knowledge, this is the first review that specifically addresses the topic of biological risk due to the monkeypox virus in occupational settings. By following the PRISMA-ScR checklist, this review provides practical implications based on the evidence present in the literature and delineates a framework of monkeypox risk, mainly in occupational settings.

## 5. Conclusions

Occupational exposure to the monkeypox virus has recently been identified as an issue of major concern for occupational health, since occupational transmission is possible and has occurred several times. Healthcare workers are considered to be at a high risk, and similar preventive measures to those adopted during the SARS-CoV-2 pandemic must be implemented in all healthcare settings. There are some recommendations for preventing and managing monkeypox in occupational settings, including the vaccination of exposed workers, the prompt identification and isolation of infected individuals, and good hygiene practices. However, until 2022, monkeypox was a rare disease, and there is limited information on the best ways to prevent and manage it in occupational settings. Education and specific training are necessary in non-endemic countries to make healthcare workers able to recognize the disease and prevent further contagions. Lessons learnt during the COVID-19 pandemic must represent a starting point to deal with this new pathogen and with new zoonotic viruses that may arise in the future. Although monkeypox seems unlikely to reach the pandemic level of spread of COVID-19, all the stakeholders must be vigilant, carefully adhering to appropriate infection control precautions. We encourage all stakeholders to leverage and improve communications, to muster resources, and to work in concert to give us the best chance of quelling this surge. We underline, once more, the need to avoid stigmatizing and discriminating against affected people and the healthcare workers caring of them, which is part of a larger narrative about global health inequities. Last but not least, we want to highlight how important it is, also in the future, to not fall into the trap of the phenomenon of "not in my backyard" in times that require us to talk about global health, after reaching globalization without fully evaluating the risks associated with it.

## Figures and Tables

**Table 1:** Ad hoc research strings used in the online search.

Database	Search String
Pubmed	“MONKEYPOX VIRUS” AND (WORKER* OR OCCUPATION*)
Scopus	(TITLE-ABS-KEY (“MONKEYPOX VIRUS”) AND TITLE-ABS-KEY (WORKER*))
Embase	(‘MONKEYPOX’/exp OR MONKEYPOX) AND (WORKER* OR ‘OCCUPATION’/exp OR OCCUPATION)
Web of Science	“MONKEYPOX VIRUS” (All Fields) AND “WORKER*” (All Fields)

**Table 2:** Key findings and practical implications of the review.

Topic	Key Findings	Practical Implications
Occupational exposure	- Occupational exposure and infections have been reported in HCWs and in workers in contact with animals in previous outbreaks; - A limited number of studies are available, despite the endemic diffusion of monkeypox in central Africa since 1970; - The monkeypox virus can remain on surfaces for a long time, but further studies are needed to detect its infection potential.	- HCWs can be considered at a high risk; - Surveillance of exposed workers for 21 days is necessary; - Exposed workers should not work with immunocompromised patients; - Evidence on the possible transmission of monkeypox to animals (domestic and farm) are missing, but in the future, other job categories may be at risk.
Preventive measures	- PPE (disposable gown and gloves, eye protection, and FFP2 masks) are needed; - Education and training are preventive measures; - Risk assessment, contact tracing, and vaccination are valid post-exposure prevention measures; - Editorials, reviews, and WHO, CDC, and ECDC guidelines provide indications about appropriate practices.	- The widespread availability of PPE is mandatory, including in outpatient clinics; - Knowledge of the disease and confidence in the diagnosis are fundamental parts of the overall prevention strategy; - As learned from the COVID-19 pandemic, training and education are measures that also protect workers’ mental health; - Vaccination with VARV vaccines, predominantly as a secondary prevention act, is effective up to 85%.
Knowledge and attitudes of healthcare workers	- Outside endemic regions, healthcare knowledge about monkeypox is limited; - The attitude towards vaccination in the selected studies on the topic is moderate; - Only a few cross-sectional studies are available on this topic.	- Specific programs of education and training for the early recognition of the disease are necessary, especially in non-endemic countries; - Awareness of the biological risk is necessary in healthcare settings to implement prevention procedures; - Programs of vaccine sensibilization may be useful.

## Data Availability

Not applicable.

## Appendix A

**Table A1:** Summary of the studies included in the review.

Reference	Country	Type of Article	Topic	Summary
Athar 2022 [26]	Iran	Editorial	Preventive measures in healthcare settings	The study is a mini-review of preventive practices for disease control in surgery rooms.
Atkinson 2022 [35]	UK	Case report	Risk of contagion in workplace	Environmental sampling was performed to identify MPXV contamination in an office where a worker infected with monkeypox had worked with evidence of low contamination.
Costello 2021 [19]	USA	Case report	Risk of contagion in healthcare settings	A total of 40 HCWs were identified as contacts. No HCW met the criteria for high-risk exposure, and no doses of the preventive smallpox vaccine were administered. Active surveillance and symptom monitoring were implemented. No disease transmission was detected.
Croft 2007 [33]	USA	Observational study	Risk of contagion in veterinarian settings	The study documented the occupational contagion of veterinary staff and other pet staff through contact with infected prairie dogs.
Dell 2020 [34]	Uganda	Cross-sectional study	Knowledge and attitudes of bushmeat traders toward monkeypox virus	Bushmeat trade workers were interviewed regarding their attitudes and practices toward zoonosis prevention. The majority believed that pathogens causing stomach ache or diarrhea (74.6%) and monkeypox (62.2%) can be transmitted by wildlife.
Doshi 2018 [29]	Congo	Case series	Preventive measures in healthcare settings	The study reported the response measures, including community education and surveillance strengthening, during a monkeypox outbreak in 2017 in the Republic of Congo.
Erez 2019 [22]	Israel	Case report	Risk of contagion in healthcare settings	A total of 11 HCWs were recognized as a monkeypox patient’s contacts; they were offered smallpox vaccination, but only 1 HCW agreed. All contacts were followed up on for 21 days; no virus transmission was detected.
Fleischauer 2005 [16]	USA	Cross-sectional study	Risk of contagion in healthcare settings	The study reported the exposure of HCWs to 3 cases of monkeypox, with at least 1 unprotected encounter. One asymptomatic HCW showed laboratory evidence of recent Orthopoxvirus infection, which was possibly attributable to either a recent infection or smallpox vaccination.
Harapan 2020a [32]	Indonesia	Cross-sectional study	Knowledge and attitudes of physicians toward monkeypox virus	Among 407 Indonesian doctors, 96% expressed acceptance of a free vaccination. Those 30 years old or younger had 2.94 times greater odds of vaccine acceptance compared to those who were older. The length of practice was also associated with the willingness of vaccination.
Harapan 2020b [30]	Indonesia	Cross-sectional study	Knowledge and attitudes of physicians toward monkeypox virus	In Indonesia, out of a total of 432 doctors, 10.0% and 36.5% of them had a good level of knowledge using an 80% and 70% cutoff point for the knowledge domain, respectively.
Hobson 2021 [21]	UK	Case report	Risk of contagion in healthcare settings	All contacts, including HCWs, identified for active surveillance completed the 21-day surveillance period from their last date of exposure and no transmissions outside the index family were identified.
Koenig 2022 [28]	USA	Editorial	Preventive measures in healthcare settings	A tool, 3I (identify, isolate, inform), was presented to manage patients with suspected and confirmed monkeypox.
Kyaw 2020 [20]	Singapore	Case report	Risk of contagion in healthcare settings and preventive measures after exposure	The level of exposure to a monkeypox patient was investigated through telephonic interviews and contact tracing. A total of 27 HCWs were identified to have had close contact, as defined by direct contact with the patient himself or the patient’s surroundings, contact with the patient’s linens, or contact with the patient’s specimens in the lab. All had protected exposure to the patient, with the appropriate and adequate use of PPE. None developed symptoms.
Lepellettier 2022 [25]	France	Review	Preventive measures in healthcare settings	Specific measures were provided for healthcare workers, including patient isolation and adequate PPE.
Nakoune 2017 [17]	Central African Republic	Case series	Risk of contagion in healthcare settings	Occupational monkeypox infections of nurses and transport clerks were reported during a familial outbreak of monkeypox in 2016 in the Central African Republic.
Nörz 2022 [23]	Germany	Observational study	Risk of contagion in healthcare settings	The study found a high viral load on several hospital surfaces that had been in contact with infected patients.
Ogoina 2019 [27]	Nigeria	Cross-sectional study	Preventive measures in healthcare settings	During the 2017 monkeypox outbreak, the hospital established a make-shift isolation ward for case management, constituted a monkeypox response team, and provided infection control resources. Training and education were major points of intervention.
Palmore 2022 [24]	USA	Editorial	Preventive measures in healthcare settings	The paper provided an overview of the fundamental preventive practices to be implemented in HCW settings.
Petersen 2019 [18]	Congo	Editorial	Risk of contagion in healthcare settings	The healthcare-associated transmission of monkeypox was observed on multiple occasions in areas where the disease is endemic, where vaccination for workers can be useful to reduce the occupational risk of disease.
Riccò 2022 [31]	Italy	Cross-sectional study	Knowledge and attitudes of physicians toward monkeypox virus	The knowledge status was quite unsatisfying, with substantial knowledge gaps on all aspects of MPX. An analysis of risk perception suggested a substantial overlooking of MPX as a pathogen.
Vaughan 2020 [15]	UK	Case report	Risk of contagion in healthcare settings	Reported the occupational contagion of a HCW from an infected patient, probably through contact with contaminated bedding.

**Table A2:** Main monkeypox management guidelines in the regions/countries where the retrieved studies were conducted.

Europe	European countries refer to ECDC guidelines in terms of prevention measures in workplaces, especially for healthcare facilities. National governments and the Health Ministry of the individual countries issued national prevention measures based on ECDC and WHO recommendations.
USA	The USA refers to CDC guidelines for workplace prevention (mainly for healthcare settings), including the use of PPE, environmental infection control, and post-exposure measures.
Asia	Asian countries have reported only a few cases during the current monkeypox outbreak so far [64]. Examples of monkeypox management guidelines are those issued in Taiwan and Singapore [65]. Guidelines regarding cases management and preventive measures have also been provided by the Saudi Arabia Public Authority [66].
Africa	An example of monkeypox management guidelines is provided by the Nigerian Centre of Disease Prevention [67]. The WHO guidelines are a major reference point.
